# Trends in Mortality From Malignant Neoplasms of the Liver and Intrahepatic Bile Ducts in the United States: A Retrospective Analysis From 1999 to 2020

**DOI:** 10.7759/cureus.95468

**Published:** 2025-10-26

**Authors:** Fawad Talat, Abdul Subhan Talpur, Hamza Usman, Manahil Rashid, Rehan Ishaque, Maryam Ali, Mansi Kallem, Madhuri Yalamanchili

**Affiliations:** 1 Internal Medicine, United Health Services (UHS) Wilson Medical Center, Johnson City, USA; 2 Pediatrics, Liaquat University of Medical and Health Sciences, Jamshoro, PAK; 3 Medicine, East Carolina University (ECU) Health Medical Center, Greenville, USA; 4 Hematology and Oncology, United Health Services (UHS) Wilson Medical Center, Johnson City, USA; 5 Medical Oncology, Broome Oncology, Johnson City, USA

**Keywords:** cdc wonder, cholangiocarcinoma, gi oncology, liver cancer, mortality trends

## Abstract

Background: Primary malignant liver tumors constitute a significant global health challenge. We conducted an analysis of mortality trends across all forms of primary liver cancers, including hepatocellular carcinoma (HCC), cholangiocarcinoma, and other less prevalent types, to enhance our understanding of the overall burden these cancers impose on the US population.

Method: The Centers for Disease Control and Prevention Wide-Ranging Online Data for Epidemiologic Research (CDC WONDER) database was analyzed, and the International Classification of Diseases, 10th Revision (ICD-10), codes were utilized to identify deaths from primary malignant neoplasms of the liver from 1999 to 2020 in patients aged 25 or older. The age-adjusted mortality rates (AAMR/100,000) for the population were extracted, and trends were analyzed for age, gender, race, and year.

Result: There were a total of 445,389 deaths from primary liver cancer from 1999 to 2020. AAMR increased from 6.95 in 1999 to 10.12 in 2020. Gender analysis showed that both sexes have shown a gradual increase in AAMR over the course of years. The mean AAMR for males was 13.09 per 100,000 population (range: 12.31-13.87; SD: 1.75), which was more than twice the rate observed in females (mean: 5.45; range: 5.16-5.74; SD: 0.66). Race analysis showed that Asian or Pacific Islanders are the only race that has shown an overall decreasing trend in mortality from liver cancer, with AAMR decreasing from 15.68 in 1999 to 12.15 in 2020. Despite the decreasing trend, Asian or Pacific Islanders still have the highest mean AAMR (mean: 14.83; range: 14.21-15.46; SD: 1.41), followed by Hispanics (13.15), Black or African Americans (11.52), American Indians or Alaskan Natives (10.51), and finally Whites (8.32).

Discussion: An important aspect of understanding the disparities in mortality from primary liver cancer will be to see these disparities in the context of HCC, which accounts for 85% of primary liver malignancies. The decreasing mortality trend from primary liver cancer observed in Asians is consistent with a decreasing trend in mortality from HCC due to a decreasing incidence of hepatitis B virus (HBV). The increased mortality from primary liver cancer among males noted throughout the study period can be attributed to higher incidences of HBV and hepatitis C virus (HCV) infections, as well as greater alcohol consumption, all of which contribute to the development of HCC. Additionally, there is evidence to support that estrogens play a protective role, limiting liver inflammation and fibrogenesis and counteracting the development of HCC in females.

Conclusion: With an increasing mortality trend from primary malignant neoplasms of the liver, further research is needed to mitigate the risk factors and advance the treatment modalities for liver cancer.

## Introduction

Primary malignant neoplasm of the liver remains a significant cause of morbidity and mortality worldwide. It is estimated that approximately 905,677 people were diagnosed with primary liver cancer worldwide in 2020 with an age-standardized incidence rate of 9.5 per 100,000 [1]. 

Primary liver cancer is in fact the sixth most common diagnosed cancer and the third most common cause of cancer-related mortality worldwide [2,3]. The two main subtypes of primary liver cancer include hepatocellular carcinoma (HCC) and intrahepatic cholangiocarcinoma. Additionally, there are other mixed types according to the pathological type [1]. An upward trend has been noted in both mortality and incidence of HCC, which is the most common type of malignant neoplasm of the liver [3]. Similarly, intrahepatic bile duct cancer (intrahepatic cholangiocarcinoma), which is the second most common primary liver cancer, is also showing an increase in mortality trend in most countries worldwide [4]. 

The main purpose of our study was to assess the mortality trends from all types of malignant neoplasms of the liver and intrahepatic ducts in the US population over the course of almost two decades from 1999 to 2020. The trends in mortality have been studied for individual types of liver cancers or bile duct cancers in the past. This study includes all types of primary liver cancers and cholangiocarcinomas as well as other rare types of malignant neoplasms of the liver to help better assess the trends in the overall burden of this group of cancers. 

## Materials and methods

The study utilized data from the Centers for Disease Control and Prevention Wide-Ranging Online Data for Epidemiologic Research (CDC WONDER) database to identify deaths associated with malignant neoplasms of the liver and intrahepatic bile ducts in the United States between 1999 and 2020. The CDC WONDER database contains information on the cause of death recorded on death certificates from all 50 states and the District of Columbia. This database has been used in various studies to assess mortality patterns over the course of almost two decades from various malignancies and other pathologies. For the purposes of this study, we utilized the following International Classification of Diseases, 10th Revision (ICD-10), codes: C22.0 (liver cell carcinoma: malignant neoplasms); C22.1 (intrahepatic bile duct carcinoma: malignant neoplasms); C22.2 (hepatoblastoma: malignant neoplasms); C22.3 (angiosarcoma of the liver: malignant neoplasms); C22.4 (other sarcomas of the liver: malignant neoplasms); C22.7 (other specified carcinomas of the liver: malignant neoplasms); and C22.9 (liver, unspecified: malignant neoplasms). The dataset was filtered to find all deaths linked to these liver cancers using these codes. The data was analyzed by age, gender (male, female), race (White, Black, Hispanic, Asian/Pacific Islander), and year.

The main outcome measure for this study was the age-adjusted mortality rate (AAMR) per 100,000 people. The AAMR accounts for the effects of population age structure and prevents the confounding of observed trends by demographic shifts over time. The Joinpoint Regression Program (version 5.2.0, National Cancer Institute, Bethesda, MD, USA) was used to find out the annual percent change (APC) in the AAMR. Joinpoint uses log-linear regression to detect significant changes in AAMR. The APC provided a better understanding of how mortality trends changed over time. This statistical method is especially helpful for identifying notable shifts in patterns over time, particularly when there are several growth or decline periods.

## Results

There was a total of 445,389 deaths from primary liver cancer in the United States from 1999 to 2020. There was an increase in AAMR from 6.95 in 1999 to 10.12 in 2020. 

**Figure 1:**
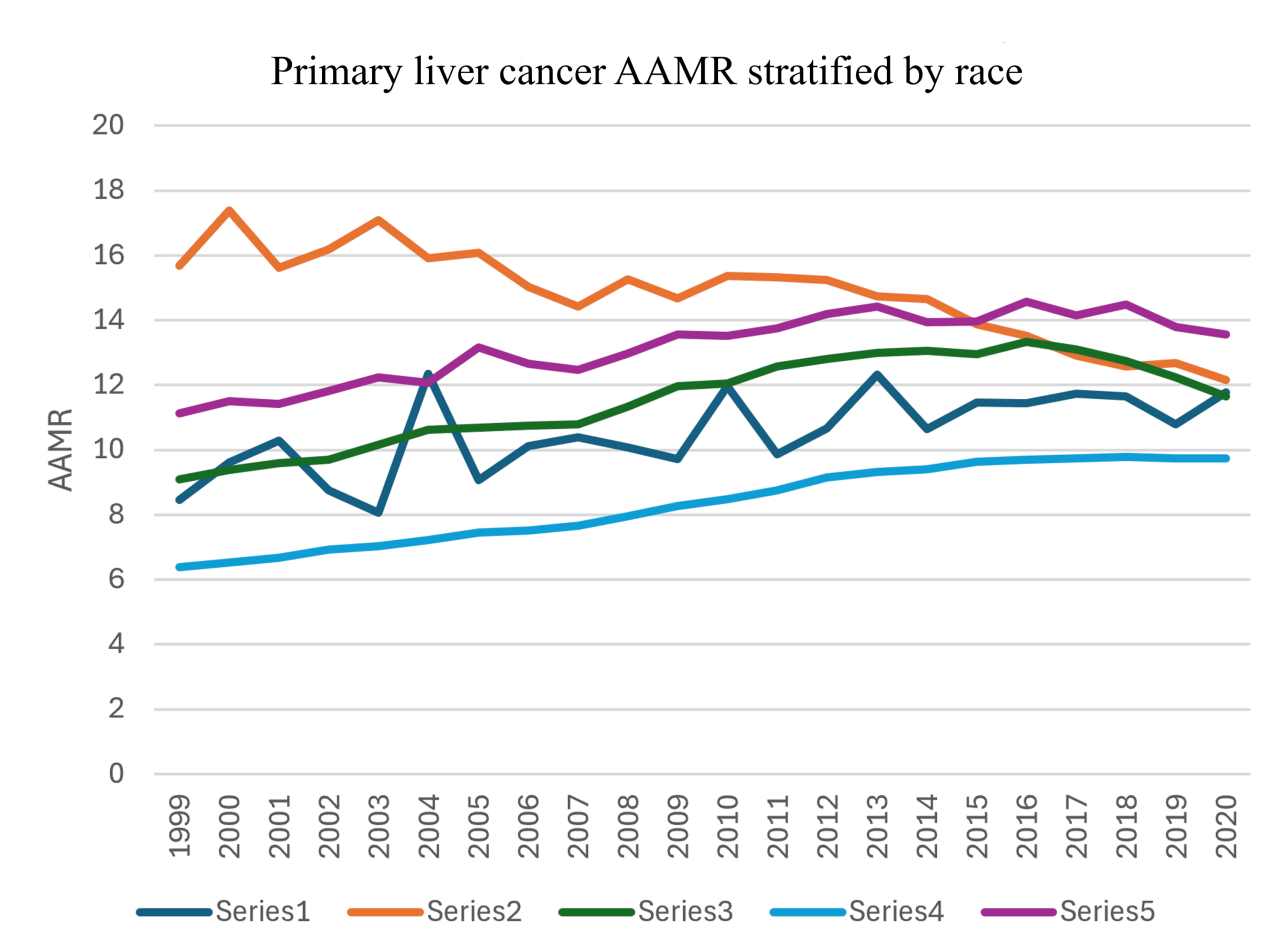
Trends in mortality stratified by race AAMR: age-adjusted mortality rates

**Figure 2:**
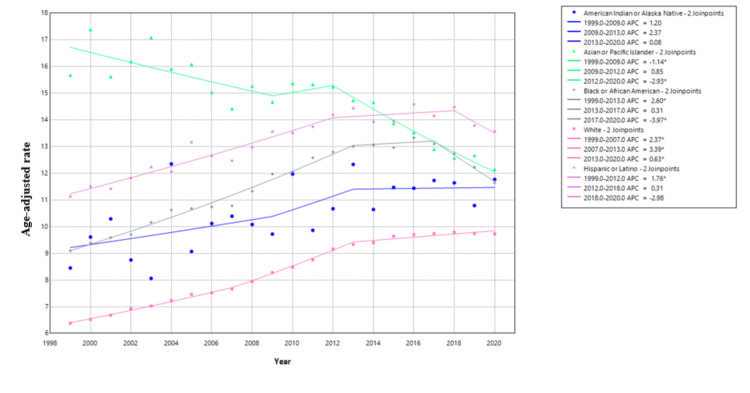
Joinpoint regression for AAMR stratified by race AAMR: age-adjusted mortality rates; APC: annual percent change

Race analysis shows that Asian or Pacific Islanders had the highest AAMR from 1999 to 2014. But the AAMR was gradually decreasing over the course of years in this time period from 1999 to 2014. From 2015 to 2020, Hispanics had the highest AAMR from primary liver malignancy. If we look at the overall trend, Asian or Pacific Islanders are the only race which have shown an overall decreasing trend in mortality from primary liver cancer. Their AAMR in fact decreased from 15.68 in 1999 to 12.15 in 2020. The highest rate was observed in 2000 (17.38), showing an overall downward trend with some fluctuations. For this subgroup, APC for AAMR was -1.14 (95% CI: -2.03 to -0.24) from 1999 to 2009 and was -2.93 (95% CI: -3.85 to -1.99) from 2012 to 2020. Whites have had the lowest AAMR, but it is consistently trending upward. Keeping fluctuations aside, Black or African Americans showed a gradual increase in AAMR from 1999 to 2016, and the AAMR is now steadily downtrending for this race group. For Black or African Americans, the APC for AAMR was 2.60 (95% CI: 2.36-2.85) from 1999 to 2013 and -3.97 (95% CI: -5.76 to -2.13) from 2017 to 2020. For American Indians or Alaskan Natives, the AAMR increased from 8.45 in 1999 to 11.77 in 2020. The peak was in 2013 at 12.33, and the lowest recorded AAMR was in 2003 (8.06), indicating a fluctuating but overall upward trend (Figure 1 and Figure 2).

The mean AAMR calculated for each race has been described in Table 1. The Asian or Pacific Islanders have the highest mean AAMR (14.83). The second-highest mean AAMR was noted in Hispanics (13.15), followed by Black or African Americans (11.52), American Indians or Alaskan Natives (10.51), and finally Whites (8.32).

**Table 1:** Mean AAMR for each race AAMR: age-adjusted mortality rates

Race	Mean AAMR	SD	95% CI lower	95% CI upper
American Indian or Alaskan Native	10.51	1.25	9.96	11.06
Asian or Pacific Islander	14.83	1.41	14.21	15.46
Black or African American	11.52	1.37	10.92	12.13
Hispanic or Latino	13.15	1.08	12.67	13.63
White	8.32	1.22	7.78	8.86

**Figure 3:**
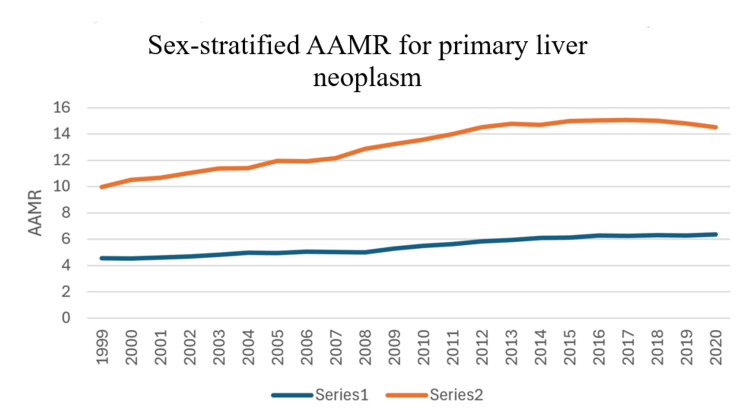
Trends in mortality stratified by sex AAMR: age-adjusted mortality rates

Both sexes have shown a gradual increase in AAMR over the course of years (Figure 3). Males have a significantly and consistently higher AAMR as compared to females. In fact, the mean AAMR for males was 13.09 per 100,000 population (range: 12.31-13.87; SD: 1.75), which was more than twice the rate observed in females (mean: 5.45; range: 5.16-5.74; SD: 0.66). This difference persisted throughout the study period from 1999 to 2020 (Table 2).

**Table 2:** Mean AAMR for sex AAMR: age-adjusted mortality rates

Sex	Mean AAMR	SD	95% CI lower	95% CI upper
Female	5.45	0.66	5.16	5.74
Male	13.09	1.75	12.31	13.87

**Figure 4:**
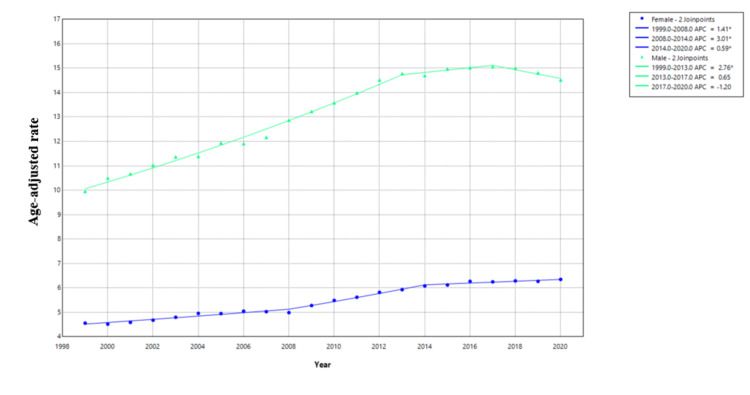
Joinpoint regression for AAMR stratified by sex AAMR: age-adjusted mortality rates; APC: annual percent change

For males, APC for AAMR was 2.76 (95% CI: 2.57-2.94) from 1999 to 2013. For females, APC for AAMR was 1.41 (95% CI: 1.11-1.71) from 1999 to 2008 and was 3.01 (95% CI: 2.37-3.65) from 2008 to 2014 (Figure 4).

## Discussion

An important aspect of understanding the disparities in liver cancer will be to see these disparities in the context of HCC, which in fact accounts for 85% of liver malignancies [3]. 

Keeping yearly fluctuations aside, most races have shown an overall increase in AAMR from primary liver cancers. Asian or Pacific Islanders are the only race group which have shown an overall decreasing mortality trend (AAMR decreased from 15.68 in 1999 to 12.15 in 2020). Despite the decreasing trend, Asian or Pacific Islanders still had the highest mean AAMR (14.83) over the course of the study period. This can be explained by the fact that Asian or Pacific Islanders represent 50% of individuals affected by hepatitis B in the United States and hepatitis B is in fact associated with 50% cases of HCC, which in turn is the most common primary liver malignancy [5,6]. The high but decreasing prevalence of hepatitis B in the Asian population and the strong association of hepatitis B with HCC explain the trends in AAMR noted among the Asian population in our study. 

The second-highest mean AAMR was noted in Hispanics (13.15), and it was followed by African Americans (11.52). Furthermore, both of these races have shown an increase in AAMR from primary liver malignancy throughout the study period. These groups are less likely to have health insurance, which limits healthcare access especially specialized treatments including the surgical removal of a tumor, liver transplantation, and ablation. There is data to support that minoritized individuals are more likely to be diagnosed with liver cancer at an advanced stage [7,8]. This serves as another reminder that systemic racism needs to be addressed and more checkpoints should be in place to prevent implicit bias. Moreover, access to specialized healthcare needs to be improved to address the racial disparities in mortality from primary liver cancer [9]. 

The finding that males have a higher mortality rate from primary liver cancer is consistent with other epidemiological studies. The higher mortality in males (mean AAMR: 13.09) as compared to females (mean AAMR: 5.45) can be explained by a number of factors. We will again assess this disparity in mortality in terms of HCC. Alcohol consumption is an important risk factor that contributes to 26% of HCC, and historically, men consume more alcohol as compared to women [10,11]. Similarly, viral hepatitis, an important risk factor for HCC, is more common in males. Increased incidence of viral hepatitis in males when compared to females is attributable to several reasons including increased exposure risk, immunological differences, and differences in response to hepatitis B virus (HBV) vaccine to name a few [11]. 

Additionally, there is more and more evidence to support that estrogen limits liver inflammation and the process of fibrosis and thus has a protective effect against HCC [12,13]. There is also evidence to support that estrogen is associated with lesser aggressiveness of liver cancer, as well as better response to treatments, lower recurrence rates, and an overall better prognosis. On the other hand, androgen promotes cell proliferation and increases the risk of developing HCC [14,15]. Further research is needed to assess if estrogen and androgen are associated with decreased or increased risk for developing other types of primary liver malignancies. 

## Conclusions

Primary liver cancer mortality has increased overall in the United States from 1999 to 2020, with the AAMR rising from 6.95 to 10.12 per 100,000 population. Significant racial disparities in liver cancer mortality are noted in the study. Asian or Pacific Islanders had the highest AAMR from 1999 to 2014, but showed an overall decreasing trend. Hispanics had the highest AAMR from 2015 to 2020. Whites consistently had the lowest AAMR, but it has been trending upward. Black or African Americans showed an increase until 2016, followed by a downward trend. American Indian or Alaskan Native populations showed a fluctuating but overall upward trend. Gender disparities are also evident in the study with males having consistently higher mortality rates than females. Additionally, both sexes showed a gradual increase in AAMR over time. These findings highlight the need for targeted interventions for high-risk racial groups and improved access to specialized healthcare for minority populations. It also underlines the importance of addressing systemic racism and implicit bias in healthcare as well as stresses the need for gender-specific prevention and treatment strategies.
